# Ventilation

**Published:** 1867-12

**Authors:** 


### ARTICLE XI.

### *Ventilation.*

Look at an asthmatic sitting before an open window, regardless of the cold, though it be winter, with his chest heaving laboriously, and his countenance expressive of exquisite anguish. What is the matter? Is he in pain? No. What, then, is the distress? It is simply from want of a due supply of fresh air. The spasm in his lungs not only prevents the free admission of air from without, but the free egress of that which is within, so that the air which is in the lungs is a mixture of foul and good air.

When so many died in the famous Black Hole at Calcutta, it was because the pure air was shut out, that they could not even get as much as the asthmatic does.

Here we have palpable results, and they startle us; and yet we may be suffering from day to day, in so small a way as to be imperceptible, the evil results of deficiency of air, which may so accumulate as to impair the health, and even perhaps ultimately destroy life. It is only a few that occasionally lose their lives suddenly from want of air, but a comparatively slight but continuous deficiency in its supply is constantly destroying vast multitudes by a slow poisoning.

A good supply of fresh air is an imperative necessity. Such a supply it is easy to get when we are out of doors; but we do not get it when we are in doors unless we make special provision for it; or, in other words, unless we take measures to secure ventilation.

A proper supply of pure air in our habitations and places of public meeting *costs something*, at least in cold weather. That is the chief difficulty. Economy is in the way. Less fuel is required with defective than with proper ventilation.

A small room closely shut up is warmed at less expense than a large room with suitable inlets for fresh air and outlets for foul.

The necessity for freeness in ventilation may be seen if we look at the amount of fresh air required for consumption. Each person requires a gallon every minute, that is, fourteen hundred and forty gallons in twenty-four hours. It is easy to see that small and closely shut-up apartments, and large gatherings of people in public buildings, as they are ordinarily constructed, are incompatible with any such supply as this.

That you may see clearly what the necessity for ventilation is, observe what the lungs actually do with the air which they receive. Pure air is composed of three gases in certain proportions: oxygen, nitrogen, and carbonic acid; this latter being in very small quantity. These proportions are altered in the lungs, so that the air which is breathed out is different from that which is breathed in. It has less of oxygen and more of carbonic acid. It is less vivifying by the loss of oxygen—that is, is thus negatively injured—and it has also acquired a positively bad character by the increase of the carbonic acid. Much increase of this renders the air palpably poisonous.

If, therefore, there be great lack of ventilation, as there often is in small rooms in dwellings, or in crowded public assemblies, much injury is done to the health by the diminution of vigor from the loss of oxygen, and by the direct poisonous influence of the added carbonic acid. And if the exposure of these deleterious influences be frequent, there will inevitably be an accumulation of evil results, seen in a broken-down system, in positive disease, and at length in death.

Observe what provision is made in nature for the constant purification of the air, and how this is often more or less defeated by the arrangements of man. As oxygen is taken up in the lungs of all animals, and carbonic acid gas is sent forth from them, breathing is continually deteriorating the air. But this is remedied by a counter operation.

Every leaf that you see is doing just the opposite of what lungs do—it takes in carbonic acid and emits oxygen—so that there is an exchange going on between leaves and lungs. In this way the due proportion of the ingredients of the air is everywhere maintained, so that if the chemist examines air taken from various quarters of the earth, he always finds precisely the same proportions.

But this is true only of air that is free, and not of that which is shut up where there are sources of contamination. Wherever there is breathing going on, if ventilation be not properly attended to there is a want of these natural proportions, and the deterioration is increased by fires and lights, for they, like lungs, use up oxygen, and return carbonic acid to the air.

There is still another important provision for the purification of air. The three ingredients of the air are not of the same specific gravity. The carbonic acid gas is decidedly heavier than the oxygen and nitrogen, and therefore has a tendency to lie below them, as water lies below oil. Now if this tendency were not obviated in some way, the carbonic acid generated from lungs and fires and various decompositions, would accumulate all over the surface of the earth, pushing up the oxygen and nitrogen above it, as water does oil, and would destroy life, and put out fires everywhere.

But this tendency is obviated by another—the tendency of gases to mingle together. It is just as the heavier water does not remain below the lighter alchohol poured upon it, but mixes with it. Agitation promotes this mingling, and therefore, in ventilation, the communication of motion to the air is an important measure, and should be accomplished so far as it can be done without inconvenience.—*London Herald.*

